# Editorial: Conservation Genomic Studies for Threatened Plants

**DOI:** 10.3389/fgene.2021.778712

**Published:** 2021-10-14

**Authors:** Xiao-Ling Tian, Yong-Peng Ma

**Affiliations:** ^1^ Guiyang Institute of Humanities and Technology, Guiyang, China; ^2^ Yunnan Key Laboratory for Integrative Conservation of Plant Species with Extremely Small Populations, Kunming Institute of Botany, Chinese Academy of Sciences, Kunming, China

**Keywords:** conservation genomics, demographic history, genetic load, threatened plants, Next generat ion sequencing

For plant species that currently have extremely small, fragmented populations, several threats can exist. These might include, but are not limited to: lower genetic diversity that can reduce the capacity to respond to environmental changes; inbreeding depression, particularly for fragmented populations, that can reduce fitness in the offspring; and genetic load that allows the accumulation of deleterious mutations (Ma et al., 2021). Such threats can be attributed to both long-term demographic events (e.g. bottlenecks, founder effects) and/or middle- or short-term anthropogenic activities. Therefore, accurate estimates of the genetic parameters mentioned above, and clear understanding of the contributing factors would ultimately improve conservation efforts to rescue threatened plants.

The eight published manuscripts covering this research topic include several types of threatened plants, from gymnosperms to angiosperms, from woody plants to grasses, and from water to land plants. Seven of these papers address conservation issues in plants with a mostly Chinese distribution, however, Brilhante et al. explored genome sizes in 24 species of *Aeonium* across the Macaronesian islands. Intriguingly, this study revealed a positive correlation between genome size and conservation status, with the more endangered species having generally larger genomes.

Three papers addressed conservation genetic questions in multiple species. Geng et al. explored factors contributing to patterns of distribution and population genetic differentiation in seven mangrove species, which are predominantly found along the coastlines of South China. The results of this study suggest that historically, populations of mangroves were more inter-connected and have recently become isolated, likely due to a combination of ocean currents and human activity. Moreover, the recent isolation coupled with lack of gene flow between mangrove populations could affect their long-term survival. Zhou et al. investigated conservation genetics in three *Salvia* species, identified centers of genetic diversity and revealed variable population structures, differing demographic histories and types of gene flow that maintain the current distribution of these species. Tao et al. performed a comprehensive analysis on six representative species of *Cycas* in South China. Their results suggested that both climate fluctuations and frequent geological activity during the late Pleistocene had a deep impact on the population dynamics of these six species. Moreover, the authors recommended several conservation actions to help rescue these *Cycas*.

The remaining four papers addressed conservation questions in single threatened but commercially important plant species. Zhong et al. genetically evaluated a total of 955 germplasms from an important medicinal and fruit crop, *Akebia trifoliata*. 164 core germplasms were proposed for further conservation and management. Qian et al. focused on a desert plant (sand rice) to explore the genomic footprints of local adaptation to ecological heterogenous habitats. The study not only revealed the basic population structure and biogeography of the species, but also provided a footprint of the local adaptations to the extreme and heterogeneous habitats in the arid and semi-arid regions of China. The study by Zhong et al. into the Munake grape provided valuable genomic resources, which should allow a deeper understanding of the genomic sequences of Munake cultivars and contribute to better knowledge of the genetic basis of its key agronomic traits. Chen et al. performed a population genomic analysis on a tiny perennial grass species with a restricted distribution. Low genetic diversity but strong population structure was revealed, indicating that populations can be effectively isolated even at small spatial scales, favoring genetic differentiation within species.

Without question, species (as the basic units for biodiversity conservation and effective conservation of threatened plants) should be a permanent topic for conservation biologists (Sun et al., 2019). Papers in this field provide many valuable insights into plant conservation and resource utilization. However, careful research addressing conservation issues with large-scale data (e.g. whole genome resequencing data) and comprehensive analyses are desirable for the future. For instance, as well as preliminary investigations into population genetics and demographic history, inbreeding depression and its associated genetic load should also be included as an important clue for predicting the fate of endangered plants and to allow conservation guidelines for their rescuing to be proposed (Yang et al., 2018). Furthermore, as whole-genome sequencing becomes more readily available, we expect more studies to use genomic data from threatened plants to investigate conservation genetics of these species and to inform conservation policy.
